# Goldilocks and the Raster Grid: Selecting Scale when Evaluating Conservation Programs

**DOI:** 10.1371/journal.pone.0167945

**Published:** 2016-12-22

**Authors:** Andre Fernandes Tomon Avelino, Kathy Baylis, Jordi Honey-Rosés

**Affiliations:** 1 Department of Agricultural and Consumer Economics, University of Illinois, Urbana-Champaign, Urbana, Illinois, United States of America; 2 School of Community and Regional Planning, University of British Columbia, Vancouver, Canada; Pacific Northwest National Laboratory, UNITED STATES

## Abstract

Access to high quality spatial data raises fundamental questions about how to select the appropriate scale and unit of analysis. Studies that evaluate the impact of conservation programs have used multiple scales and areal units: from 5x5 km grids; to 30m pixels; to irregular units based on land uses or political boundaries. These choices affect the estimate of program impact. The bias associated with scale and unit selection is a part of a well-known dilemma called the *modifiable areal unit problem* (MAUP). We introduce this dilemma to the literature on impact evaluation and then explore the tradeoffs made when choosing different areal units. To illustrate the consequences of the MAUP, we begin by examining the effect of scale selection when evaluating a protected area in Mexico using real data. We then develop a Monte Carlo experiment that simulates a conservation intervention. We find that estimates of treatment effects and variable coefficients are only accurate under restrictive circumstances. Under more realistic conditions, we find biased estimates associated with scale choices that are both too large or too small relative to the data generating process or decision unit. In our context, the MAUP may reflect an *errors in variables problem*, where imprecise measures of the independent variables will bias the coefficient estimates toward zero. This problem may be pronounced at small scales of analysis. Aggregation may reduce this bias for continuous variables, but aggregation exacerbates bias when using a discrete measure of treatment. While we do not find a solution to these issues, even though treatment effects are generally underestimated. We conclude with suggestions on how researchers might navigate their choice of scale and aerial unit when evaluating conservation policies.

*"This porridge is too hot!" exclaimed Goldilocks*.

*So, she tasted the porridge from the second bowl*.

*"This porridge is too cold," she said*.

*So, she tasted the last bowl of porridge*.

*"Ahhh, this porridge is just right”*.

## 1. Introduction

Spatial data on land use or forest cover is a key ingredient for studies seeking to measure the impact of conservation programs [1,2]. We have witnessed vast improvements in the quality of remote sensed imagery in the last decade, and it is now common to analyze spatial data obtained at resolutions of less than 1 m per pixel [3]. These high quality data provide researchers with new opportunities to monitor and quantify the impact of conservation programs such as protected areas, payment for environmental services (PES) programs or forest management practices.

In this paper, we examine how the choice of scale influences estimates of program impact when evaluating conservation programs. Until now, the scale and structure of spatial data has been taken as given [4,5] and estimates of conservation programs have used units of analyses that range from small pixels to 5x5 km grids (Table 1). In only a few instances have researchers examined conservation impacts at multiple scales [6,7], suggesting that for the most part, analysts implicitly assume that choices about scale will not affect estimates of treatment effects for conservation programs. When access to spatial data was limited, this was the only assumption possible. However, researchers now have access to multiple data sources at very high resolutions. For instance, the resolution of satellite imagery is now so good, that in temperate forests, one can identify the bare ground between the trees and through the branches, thus literally, losing sight of the forest within the trees. At least in terms of forest cover, we have reached the maximum resolution necessary, since, for the purposes of measuring forest change, higher resolution data will not provide better estimates. In this new technological landscape, researchers will need to think critically about the scale at which conservation decisions are made and accordingly choose the appropriate scale and areal unit of analyses. These choices present researchers with well-known challenges related to scale, zoning, and spatial autocorrelation [8–10].

**Table 1:** Comparison of methods and scale used in studies that evaluate conservation impact. (T: # treatment units; C: # control units).

Authors	Zoning	Cell size (ha)	Study Area (km^2^)	# Obs.	Imagery & Resolution	Method	Sample	Spatial
Alix-Garcia et al. 2012 [28]	Grid	6	335,000	633	MODIS (250 m)	Matching	Yes	No
Andam et al. 2008 [29]	Grid	3	30,357	15,383	Landsat TM (30 m)	Matching	Yes	No
Baylis et al. 2015 [24]	Grid	4	3,427	85,693	Landsat TM (30 m)	Matching	No	Yes
Blackman 2015 [30]	Grid	6.25	21,000	397,376	Landsat (30 m)	Matching	Yes	No
Börner et al 2015 [7]	Grid	40,000	4,100,000	11,181	Landsat (100 m) & MODIS (250 m)	Matching	No	Yes
Bruggeman et al. 2015 [31]	Grid	0.09	24,000	3,000	Landsat ETM (30 m)	Matching	Yes	No
Busch & Grantham 2013 [32]	Grid	900	1,759,194	195,466	MODIS (250 m)	OSIRIS model	No	No
Clements & Milner-Gulland 2014 [33]	Grid	100	3,304	3,304	Landsat TM (30 m) Aster (30 m)	Matching	Yes	No
Costedoat et al 2015 [6]	Grid	10	1,468	2,524	SPOT 5 (10 m)	Matching	No	Yes
Ferraro & Hanauer 2014 [34]	Object: Census Boundaries	295 (mean)	50,660	17,239	Landsat TM (30 m)	Matching	No	No
Gaveau et al 2009 [35]	Grid	2	440,000	1,256 (T)	Landsat TM (30 m)	Propensity Score Matching	Yes	No
Honey-Rosés et al 2011 [17]	Object: Landscape units	20.81 (mean) 5.44 (median)	3,432	4,263 (T+C) 425 (T)	Landsat TM (30 m)	Matching	No	Yes
Pfaff 1999 [36]	Object: County	33,000 (mean)	5,000,000	480	Landsat TM (reclassified to 1 km^2^)	Regression	No	No
Pfaff et al. 2015 [37]	Grid	0.81	156,522	20,072 (T+C) 7,775 (T)	Landsat TM (30 m)	Matching	Yes	Yes
Sanchez-Azofeifa et al 2007 [4]	Grid	2,500	50,100	2,021	Landsat TM (30 m)	OLS	No	No
Shah & Baylis 2015 [38]	Grid	900	1,565,604	3,957(T) 170,899(C)	MODIS (250 m)	Matching	No	Yes
Sills et al 2015 [39]	Object: Municipality	NA	15,000	NA	INPE Brazilian National Space Agency	Synthetic Control	No	No
Sims 2010 [40]	Object: Locality	8,200 (mean)	147,620	4,113	Landsat (30 m & 60 m)	OLS & IV	No	No
Sims & Alix-Garcia 2015 [41]	Object: Thiessen polygons of locality	215 (mean)	335,000	59,536	Landsat TM (30 m)	Matching	Yes	No

The theoretical challenges that confront researchers when making decisions about scale and unit of analysis have been studied for decades by geographers and spatial statisticians [11–13]. On the one hand, it is well known that spatial data is subject to a *scale effect*, in which the observation of outcomes may differ at different levels of aggregation [13]. The scale effect results from the smoothing that occurs when moving to a higher level of aggregation, and the associated loss of heterogeneity and variation. In addition to the scale effect, the errors or bias generated by the choice of the unit of analysis are known as the *modifiable areal unit problem* (MAUP) [5,8,14]. Bias in the MAUP can result from both scale and zoning effects and their interaction, where zoning refers to alternative groupings of data at the same scale. Although recognized and extensively discussed in the aforementioned literatures, the errors and biases associated with the MAUP have yet to receive the attention they deserve within conservation science.

Spatial statisticians in the 1930s first pointed out the inaccuracies and biases associated with analyzing a spatial data set with different aerial units and scales [13]. The concept was brought to the fore by Gehlke and Biehl [10] even though the most cited evidence of the MAUP is given by Openshaw and Taylor [11], who show that depending on the choice of scale and zoning, the same data could generate correlation coefficients ranging from -0.99 to +0.99. In other early work using different aggregations of existing data, the MAUP was thought to produce unpredictable results, with little generalizable solutions. In these earlier treatments of the MAUP, the problem was essentially seen as one of aggregation: the true decision-making unit was the individual, but the data were only available aggregated, such as a census block or county [8]. In this context, the MAUP reflects the *ecological fallacy* in which it is erroneous to assume that outcomes at one scale will extrapolate accurately to finer resolutions. Recently, though, research has focused on fine scale datasets that exhibit spatial correlation, as well as how multivariate models behave under the MAUP [5,14–16]. The literature has also moved in the direction of Monte Carlo experiments using artificial data, to isolate the sources of errors and biases [16].

Work on impact evaluation in conservation has been slow to account for either scale effects or spatial dependence. While many studies that evaluate the impact of conservation programs use spatial data, few account for spatial processes (exceptions include [17–20]). Deforestation and other land use patterns are highly spatially correlated, yet limited works have sought to control for how the conditions in neighboring areas might change the land use in the target area. Controlling for spatial processes can substantively alter the estimated effect of conservation programs [17]. While spatial processes are not needed for the MAUP to generate biased estimates, they do complicate the aggregation problem.

In this paper, we build on the existing insight developed by spatial statisticians in order to assess how a researcher’s choice of scale or aerial unit may bias their estimates of program impact, or treatment effects, when evaluating conservation programs with common statistical methods. We make several contributions. First, we contribute to the conservation literature by identifying the effect of the choice of scale and spatial correlation on the estimates of conservation program efficacy. Second, we contribute to the literature on the MAUP by considering spatial processes generated at lower resolutions than the scale in which data is available to the researcher, reflecting the detailed spatial data now available to researchers.

To illustrate the sensitivity of statistical analysis to a researchers choice in scale, we begin with an analysis of deforestation data in a protected area in Mexico. We estimate the effect of the protected area using a difference-in-difference (DID) framework, comparing forest cover before and after, inside and outside the protected area. This analysis is repeated for four different levels of aggregation: one hectare areal units, 4 hectare units, 16 hectare units and 100 hectare units. The results indicate that even using a relatively conservative estimation strategy, estimates of program effectiveness consistently become less precise and increase with aggregation, growing by almost 25%.

Then, to probe why our estimates vary with scale, we develop a Monte Carlo simulation of a conservation program and observe how estimates change at different scales relative to the data generating process. We generate data at several scales, pixilate these data to represent satellite imagery, and then regress these data at different levels of aggregation to explore how aggregation, disaggregation and spatial correlation affect the coefficient estimates. Under this controlled environment, we can isolate the sources of bias and inefficiency by gradually introducing spatial correlation within and between the variables. We focus on Ordinary Least Squares (OLS) estimates, but also show similar results for difference in differences approaches and spatial lag models.

Our results highlight the problems with using units of analysis that are either too small or too large. When using a smaller spatial unit than the true scale of the process, unnecessary noise is introduced in the analysis, which bias coefficient estimates toward zero, in what is known as *an error in variables* problem. For example, imagine that a farmer is considering converting forested land to seasonal agriculture, and the main criteria for selecting the forest plot to deforest is accessibility and slope. The scale at which the farmer is making this decision is much larger than the scale at which spatial data may be obtained. LiDAR (Light Detection and Ranging), for example, may reveal slopes at the decimeter scale, including variation induced by rocks and logs. These high-resolution data, while accurate, are unlikely to determine the probability of deforestation in any 1m by 1m quadrant, and therefore will only add noise to the analysis, resulting in an attenuated estimate of the effect of slope on deforestation. Only when slope is measured at the appropriate scale, is the relationship between slope and deforestation likely to emerge. Although aggregation mitigates this problem, it introduces inefficiency to the estimates. Aggregation can also generate an error in variables problem when treatment, or the conservation intervention, is measured as a binary variable. As the definition of the treated area becomes more coarse, treatment is increasingly measured with error, leading to biased estimates of treatment effect. Moreover, one cannot assume that these errors always result in attenuated coefficient estimates. If the covariates are correlated, and they exhibit different levels of noise, the coefficient of the covariate measured with less error may be overestimated. Finally, in spatial datasets, an unaccounted spatial lag process will induce an additional bias via an omitted variable. Note that these issues arise simply from the nature of the spatial data, and before introducing behavioral effects such as leakage.

In the next section we illustrate the challenges and errors associated with scalar mismatch through an empirical example with real deforestation data. We then introduce the methods used for the experimental Monte Carlo simulations, followed by results and discussion. We end the paper with a few lessons learned and best practices.

## 2. Empirical Example: Examining the Impact of a Protected Area in Mexico

We begin our paper with a brief example using real deforestation data from a protected area in Mexico. We use this example to illustrate the range of treatment effects that one might estimate when using the same data, but changing the scalar unit of analysis. We aim to estimate the treatment effect of a protected area in reducing deforestation in the Monarch Butterfly Biosphere Reserve. The forests of this protected area serve as winter habitat for the monarch butterflies (*Danaus plexippus*) since they provide the migratory butterfly with the unique climatic conditions necessary for winter survival [21]. To protect this natural wonder, the Mexican government first established the protected area in 1986 [3].

**Fig 1:**
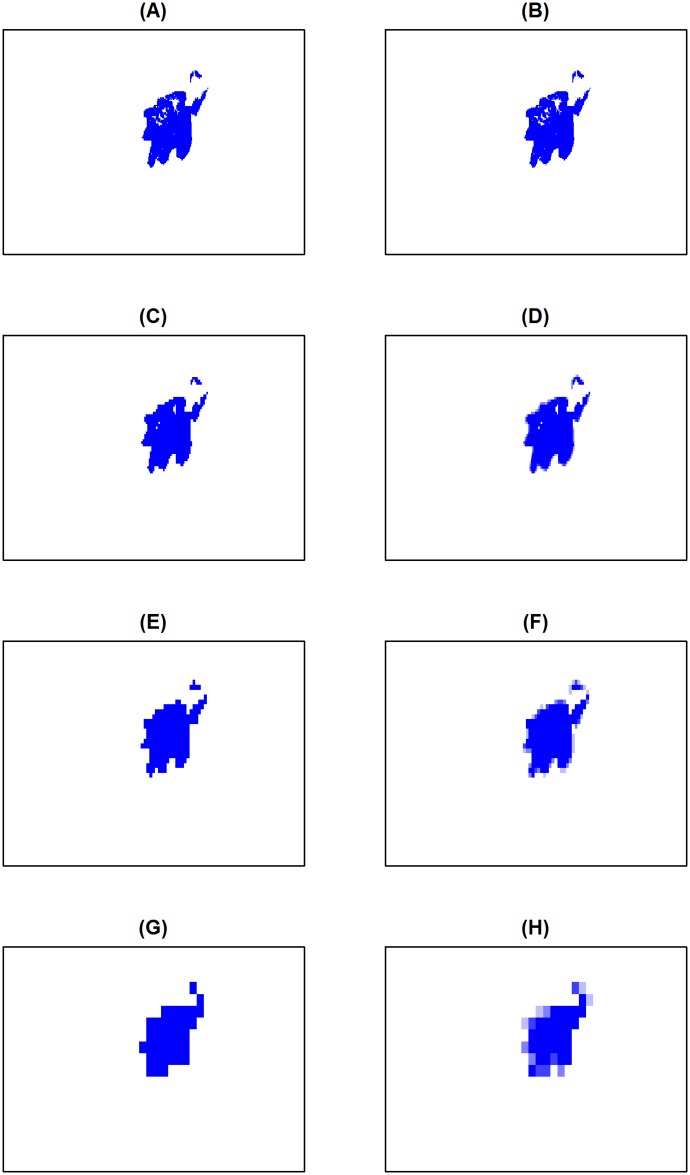
Continuous versus discrete data aggregation. Treatment (blue) units at the protected area observed at different scales (full resolution at 182 ha x 417 ha). Binary aggregation is illustrated on the left panels and continuous aggregation on the right. (A)-(B) No aggregation. (C)-(D) 2x2 Aggregation. (E)-(F) 4x4 Aggregation. (G)-(H) 10x10 Aggregation.

To evaluate the effect of legal protection in reducing deforestation, we estimate a linear panel regression with a difference-in-differences approach (also known as before-after-control-intervention or BACI). We use forest cover data generated from Landsat (30 m) taken in 1986, 1993, 2000, 2003, 2006 and 2009, where 1986 is the pre-treatment observation and the other 5 years are post-treatment, and the protected area is the intervention, and surrounding areas are the control [22]. We first use a discrete measure of treatment, defined as 1 if the majority of the unit is inside the protected area and 0 otherwise (Fig 1A, 1C, 1E and 1G). Because our areal units do not perfectly overlap the protected area, we also use a continuous measure of treatment, which measures the percent area inside the protected area (Fig 1B, 1D, 1F and 1H).

We estimate the effect of protection on percent forest cover with parcel-level fixed effects that capture time invariant parcel characteristics, such as slope and distance to road, and year fixed effects to control for common shocks, such as changes in forest product prices or labor availability. To mimic other impact evaluations of conservation policy, we also estimate the difference between forest cover in the protected area and in neighboring parcels in 2000, as an ex-post cross sectional analysis. Four levels of aggregation are considered: 1 hectare unit (1x1), 4 hectares (2x2), 16 hectares (4x4) and 100 hectare parcels (10x10) (Fig 1). Each hectare contains approximately 9 pixels from the raw land cover data. Thus, even our smallest chosen areal unit of 1 hectare aggregates above the pixel level of its satellite imagery. Note that in this example, we ignore the potential for forest leakage (Tables 2 and 3).

**Table 2:** Variation in treatment effects estimated with different methods and scales.

Observation size	(1) 1 ha	(2) 4 ha	(3) 16 ha	(4) 100 ha
DID with parcel FE, continuous treatment	0.0805***	0.0812***	0.0875***	0.0999*
	(0.00684)	(0.0130)	(0.0244)	(0.0593)
DID with parcel FE, discrete treatment	0.0805***	0.0791***	0.0836***	0.0773
	(0.00684)	(0.0125)	(0.0227)	(0.0545)
Ex-post cross section, continuous treatment	0.0841***	0.0905***	0.102***	0.135
	(0.00990)	(0.0187)	(0.0351)	(0.0859)
Ex-post cross section discrete, discrete treatment	0.0841***	0.0892***	0.0972***	0.0869
	(0.00990)	(0.0181)	(0.0326)	(0.0796)
Year Fixed Effects	yes	yes	yes	yes
Observations for diff-in-diff	48,882	12,486	3,126	492
Observations for cross-section	8,147	2,081	521	82
Standard errors in parentheses				

*** p<0.01,

** p<0.05,

* p<0.1

**Table 3:** Percent difference in coefficient estimates between discrete and continuous and over aggregation.

Observation size	(1) 1 ha	(2) 4 ha	(3) 16 ha	(4) 100 ha	(5) % Difference from small to large aggregation using continuous treatment	(6) % Difference from small to large aggregation using discrete treatment
diff-in-diff	0	2.65	4.67	29.24	24.10	4.14
ex-post cross section	0	1.46	4.94	55.35	60.71	3.22

The first two rows of Table 2 present the coefficient estimates of the treatment effect of the protected area using the difference-in-differences approach, with the first row using a continuous measure of treatment and the second row using a discrete measure. Rows 3 and 4 replicate the same analysis but use only a cross-section of data on forest change from 2000. As one moves from left to right, the unit of analysis increases from 1 ha to 100 ha parcels. The estimated coefficients for percent forest cover increase with aggregation when a continuous measure of treatment is used. This pattern holds for both the difference-in-difference estimates and the cross-sectional estimates. This pattern is less clear when discrete measures of treatment are used: except at the smallest level, the estimate from the discrete measure is smaller than the estimate from the continuous measure, and this gap increases with aggregation. This potential attenuation bias suggests an errors in variables problem which is exacerbated by using a discrete measure. As expected, the standard errors of the estimates increase due to a shrinking number of observations, rendering the estimates insignificant at the highest level of aggregation.

The percent differences in the estimates are presented in Table 3. The first 4 columns list the percent difference in the estimates of continuous versus discrete measures of treatment at the four different levels of aggregation. The second last column gives the percent difference between the smallest to largest aggregation in the estimates of the continuous measure of treatment, and the last column repeats this measure for discrete treatment. As expected, the bias generated by using a discrete measure of treatment increases with the level of aggregation, growing to 29% and 55% for the difference-in-difference and cross-sectional estimate respectively. Notably the distortion is worse for the cross-sectional analysis, possibly due to the smaller number of observations and the decreased number of controls. Second, the changes in estimates with aggregation are substantial. For the difference-in-difference estimates, the 100 ha level of aggregation produces an estimate that is 24% higher than the analysis at 1 ha, and for the cross-section, this difference is even larger, at over 60%. These differences are much smaller with discrete treatment, at 4% for the difference-in-difference estimates and 3% for the cross-sectional estimates. We believe that these smaller differences result from offsetting attenuation biases, arising from errors in variables from using noisy data at a small scale, and a second errors in variables arising from aggregating discrete treatment measures at the higher scales.

In short, we find that the estimates of the effect of the protected area vary with the chosen scale of analysis. Which scale is correct? One approach for choosing the ideal scale for analysis is to select the one that matches the decision-making unit. However, often the precise decision-making unit is unknown. In the example above, using information from detailed community forest management plans, we observe that forest management decisions are typically made on parcels of approximately 4 ha, suggesting that might be the appropriate unit of analysis.

At the same time, we must consider that the drivers of deforestation may not occur at one single scale but rather at multiple scales simultaneously [8]. The same is true for most ecological processes. Forest loss is the outcome of micro-pressures operating at fine scales–like the farmer who removes dead trees for fuel wood–, and simultaneously the result of pressures operating at large scales–like the volcanic activity that left traces of fertile soil on a landscape. This problem is compounded when multiple decision-making agents operate at multiple scales. Selecting a scale at which resources are traditionally managed, such as property boundaries, may be a useful starting point. But even then, this scale may hide tradeoffs and dynamics within the property boundary itself, especially if the area is managed collectively and there are intra community tradeoffs that prioritize the protection of some community forests over others [23]. In other words, the process that our model attempts to explain (deforestation) is the combined outcome of explanatory processes (independent variables) operating at different scales. And even closely related processes may have vastly different scales. For example, deforestation decisions may likely be made at a larger scale than reforestation. Thus, the scale issue is not merely one of limiting aggregation, but finding the appropriate unit at which to model the desired phenomenon.

## 3. Methods and Data

To study how choices pertaining to scale and areal units influence estimates of conservation program effectiveness, we employ a set of Monte Carlo simulations to generate and analyze data at multiple scales. In contrast to the previous literature that uses simulated data to explore the effect of aggregation on correlations [5, 14], in this paper we analyze the sources of the MAUP when data are both aggregated and disaggregated from the level of the true data generating process. We feel that this approach can provide more insight to the conservation literature and more accurately reflects the reality of researchers using increasingly fine-grained spatial data. We study the impacts of the choice of scale, coupled with misspecification of the spatial unit of observation, when estimating treatment impacts. Given the complex relations that exist in real datasets, we focus on simulated data to control its properties and be able to compare how different attributes of the data may lead to biased and/or imprecisely estimated coefficients.

### 3.1. Basic Setup

Consider a forest manager who determines what areas to log at a one hectare plot level, based on plot-level characteristics. Denote this scale as the “true level”. The researcher, however, has data available at a finer resolution and has to decide how to aggregate them for the analysis. Our approach allows us to capture the reality of researchers who aggregate up to their chosen unit of analysis from pixel level detail in satellite imagery or other remote sensed data. Therefore, we assume that these “observed data” are always at the pixel level, a 1x1 cell laid in a regular lattice of dimension 120x120 (*n* = 14,400).

The observed data emulate the characteristic ‘noise’ embedded in fine-resolution satellite images. In them, when determining values at the pixel level, some information, such as elevation, is generated either by an implicit form of kriging, or by direct observation. Other imagery interpretation groups land cover categories to contiguous collections of pixels, and assigns a homogenous land use cover to all pixels within this patch. Note that this process smoothes away heterogeneity, where this heterogeneity may either reflect true variation in the dependent variable or be spurious. This same smoothing does not necessarily occur for other independent variables.

**Fig 2:**
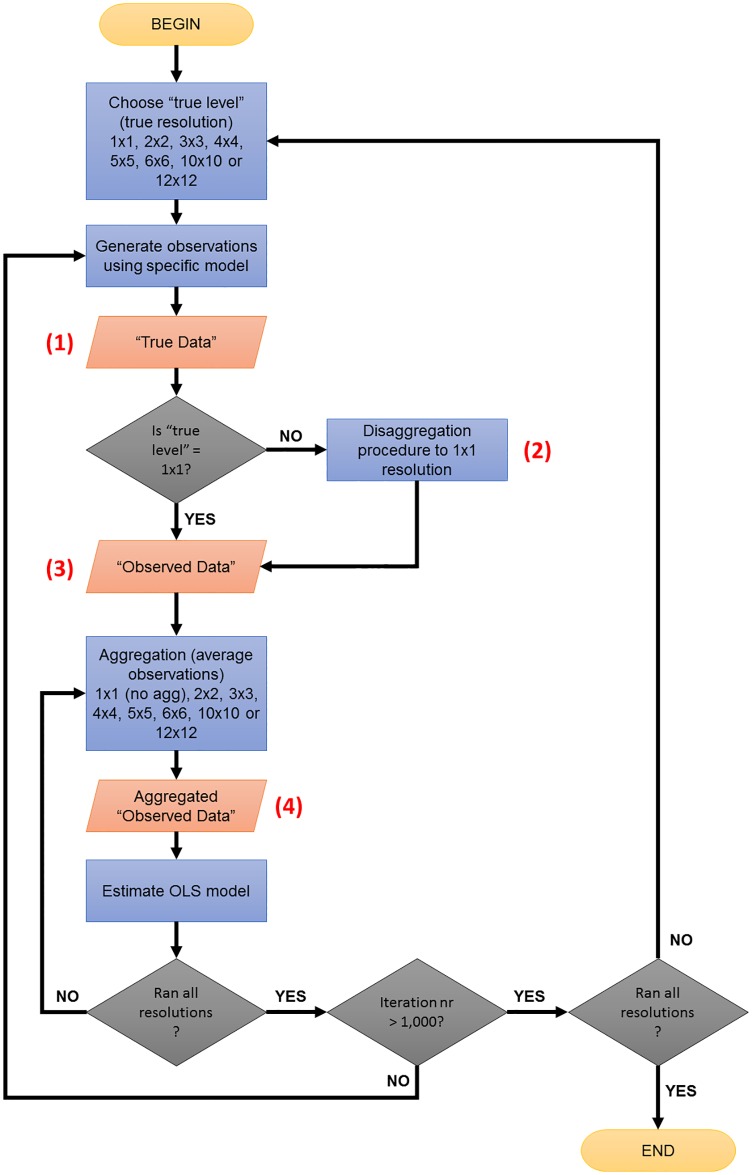
Monte Carlo Simulation Flow Chart.

**Fig 3:**
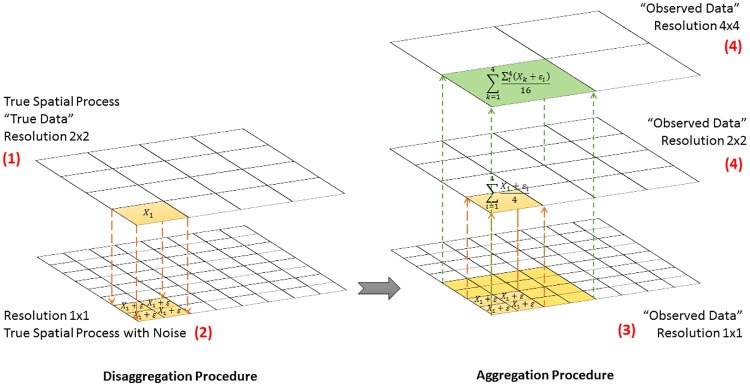
Aggregation and Disaggregation Procedure. Example of a spatial process with true level = 4x4 resolution.

For the simulation (Fig 2), observed data are derived from the spatial process at its true level disaggregated to the pixel level via a procedure that adds noise. Hence, the only instance in which ‘observed’ are equal to ‘true’ data (i.e., they do not carry any extra noise) is when the simulated spatial process is at the pixel level. In formal terms, for true levels at lower resolution than the pixel level, variables in the observed data are generated by drawing from a normal distribution with mean equals the true variable and a pre-specified variance $\sigma_{d}^{2}$ (Fig 3). Notice that by changing $\sigma_{d}^{2}$ we can simulate more or less information loss due to (dis)aggregation by imposing more spatial heterogeneity at the pixel level.

This procedure implies that the researcher never observes the actual spatial process, only a pixilated version thereof, where even if the researcher aggregates the observed data to the true level, it will not exactly match the true data. Even if the boundaries of the decision-making unit perfectly corresponded to the pixel grid, one might expect to see more heterogeneity at the pixel level than at a more aggregated level of the true DGP. If the true data generating process occurs at the pixel level, this heterogeneity may reflect the ‘truth’, whereas if the true decision-making process is at a higher level, this heterogeneity may merely be added ‘noise.’

For simplicity, we assume the researcher can choose between six possible resolutions besides the pixel level (1x1 resolution). We average the observed pixel data into groups of: 4 pixels (2x2 resolution), 16 pixels (4x4 resolution), 25 pixels (5x5 resolution), 36 pixels (6x6 resolution), 100 pixels (10x10 resolution) and 144 pixels (12x12 resolution). The scale used for analysis is assumed to be the choice of the researcher.

### 3.2. Data Generating Process

To understand the sources of the MAUP in studies that estimate treatment effects for forest conservation programs, we generate spatial processes with various degrees of complexity by altering the coefficients and spatial relations in the following generic model:

Y=α+βX+ρWY+γT+ ε

where Y is a continuous variable that represents the outcome of interest, X is an independent variable that affects outcome, and T is a dummy for treated cells, such as a conservation program. We let α = β = 1, and set the coefficient on treatment, *γ* = 0.5. Because of the high degree of spatial correlation in many land-use processes, we allow for the addition of a spatial lag process in our simulated data. Thus we assume that the outcome of interest, Y, may be a function of outcomes in contiguous parcels, where those contiguous parcels are identified through a spatial weights matrix *W*. The dependent variable Y can be determined exclusively by X and T (*ρ* = 0) or complemented by a spatial lag (*ρ* > 0). The spatial lag could result if deforestation in one cell increases the probability of deforestation in the neighboring cells, over and above the effect of independent variables. The remaining error is normally distributed: *ε* ~ *N*(0, 1).

**Fig 4:**
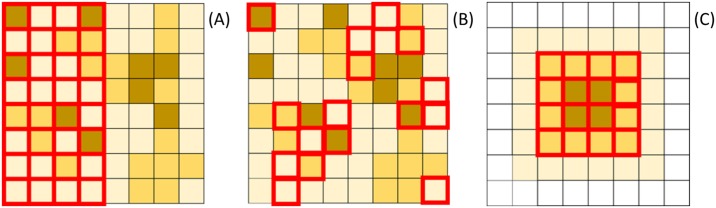
Three possible treatment scenarios. Treatment cells are identified with a red border, while the value of the independent variable X is represented by the cell color gradient (higher values for darker cells).

Treatment is represented by an indicator variable, where *T*_*i*_ = 1 implies that the unit *i* is treated. During the aggregation or disaggregation process, a unit is assigned to treatment if the majority of the pixels in the unit are treated. We allocate treatment by (1) clustering treatment cells in the Eastern segment of the grid (as in [16]) (Fig 4A); (2) allocating treatment cells randomly (Fig 4B), or (3) correlating treatment with X, in which the largest X values are treated (Fig 4C). The first scenario of clustered treatment cells resembles a wildlife preserve that is allocated over a contiguous space. The second scenario might reflect an increase in enforcement of forest use regulations where that increased enforcement is distributed randomly across space. The third scenario reflects a conservation program targeting species habitat, such as areas over a certain elevation. This last scenario provides us with a treatment allocation procedure that lies between clustered and random.

Given that factors that affect land use outcomes such as slope, elevation or soil type themselves may follow a spatial process, we also allow the independent variable X to both be random (*X* ~ *Unif*(0, 5)), or spatially correlated in two ways: *X* = (1 –*πW*)*K or X* = *W*^2^*K*, with *K* ~ *Unif*(0, 5). Therefore, we study two types of spatial dependence in the dependent variable, X: a spatial lag (*π* = 0.9) and a spatial dependence on second order neighbors (*X* = *W*^2^*K*). A spatially dependent X might result if the independent variable is generated by kriging, or simply if it is a continuous measure over space such as soil quality or slope. The two spatial processes used to generate X were chosen to reflect the impact of using an aggregation procedure that follows the spatial grid pattern (first case) or not (second case). The latter is closer to reality where the spatial process is usually unknown and a standard aggregation procedure is used, introducing zoning effects. In real data, one might expect spatial processes present in both dependent and independent variables.

### 3.3. Simulations

**Fig 5:**
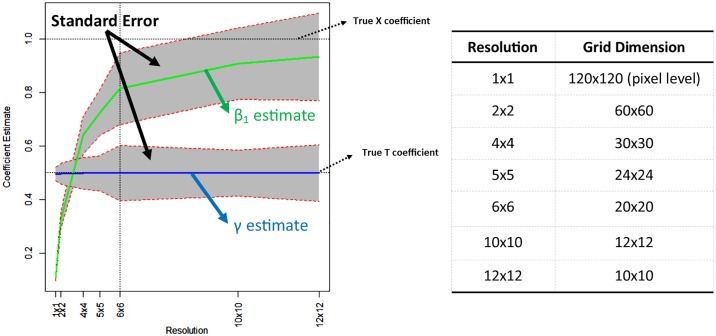
Sample chart and conversion table for the horizontal axis.

To isolate the sources of biases/inefficiencies in estimation, we generate data from five models in increasing level of complexity. A Monte Carlo with 1,000 simulations is performed for each model and resolution. The results of aggregating the observed data to different resolutions are illustrated by mapping the estimated coefficients and their standard errors against their values at the true level. In all figures that follow, the vertical axis represents the magnitudes of the coefficient estimates and the horizontal axis represents the resolution chosen (Fig 5). Thus, it ranges from the pixel level, up to the highest aggregation level of 12x12 (the lowest resolution). The vertical dashed line denotes the true level, which in the case of Fig 5 case is a 6x6 resolution. The top green line represents the coefficient estimate on X and the grey bounds represent the standard errors at each level of aggregation. The blue line represents the estimated coefficient on treatment, and again the grey area represents the standard errors of the estimate at each level of aggregation.

## 4. Results

A full explanation and detailed results for all models can be found in the Supporting Information. Here we focus on the evidence behind a few general findings:

1. Coefficient estimates may be imprecise when the analysis is at too large a scale (Model 1);
2. Coefficient estimates may be biased when analysis is done at too small a scale, and this effect is exacerbated when a spatially-correlated X is present (bias is essentially resulting from an *errors in variables problem*, which leads to attenuated estimates of both *β* and *γ*) (Models 1 and 4);
3. The aggregation problem can generate bias when one uses a discrete measure of treatment (Model 3);
4. When treatment is correlated with other covariates, as is often the case in conservation, estimates of the coefficients may be biased either upward or downward, depending on which variable becomes less precisely, treatment or X (Model 4);
5. Even an analysis performed at the level of the data generating process (DGP) may not generate consistent results (Models 2 and 4);
6. Last, a spatial lag will exacerbate bias if unaccounted for (Model 5).

In sum, efficiency issues arise when an analysis is conducted at a coarse scale due to a diminished number of observations. But bias may arise when using too small or too large a scale from an error in variables issue caused either by noise being added to the data when the unit is too small, or by the spatial structure of the variables being distorted when the unit is too large. Omitted variables enhance the previous bias. In the next subsections, we detail the key sources of the aforementioned issues, starting with a simple data process and OLS estimates until complex data and spatial econometric techniques.

### 4.1. Model 1: OLS with random covariates

**Fig 6:**
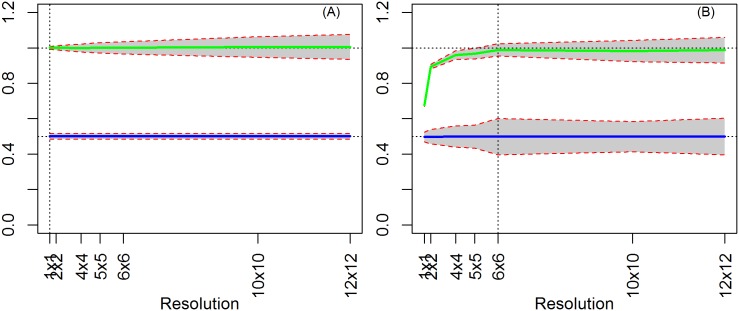
OLS results (X random, T contiguous, ρ = 0). (A) True level = 1x1 resolution (pixel level). (B) True level = 6x6 resolution.

To demonstrate the problem of using units of analysis that are too large, we show results from a simple OLS estimate in which treatment is contiguous (clustered) and the independent variable is random. We start by assuming the true level as the pixel level, i.e., “observed data” is equal to “true data” (there is no extra noise). Under these assumptions, our results are unbiased for both X and T regardless of the data's aggregation level (Fig 6A).

This first model, although improbable in the conservation context, represents the standard setting for studying the MAUP in the literature. We reproduce the results found in several earlier papers that under specific conditions, where we are able to accurately identify the coefficients on T and X [5, 14]. However, note that when the analysis is performed at higher aggregation levels, the standard errors increase monotonically due to the efficiency loss from the reduced variability and lower number of observations. Thus, using a large unit of aggregation reduces the efficiency of the estimates.

Next, we repeat the exercise assuming the true level at a higher aggregation, i.e., the “observed data” is different from the “true data”. We now have a pixel level dataset with ‘noise’. Estimates of the treatment effect (γ) remain unbiased because treatment units are spatially clustered, and thus different levels of aggregation do not affect its measure. Estimates of the coefficient on X, however, are underestimated when our chosen scale of analysis is smaller than the data generating process. This is because at a finer resolution, the estimate is sensitive to the noise added in the data, and the estimate moves toward zero as expected with an *error in variables* problem. In contrast, aggregation attenuates this bias by smoothing the additional variability, so only an efficiency loss remains when the analysis is performed at higher levels of aggregations (Fig 6B).

### 4.2. Model 2: OLS with spatially correlated X

**Fig 7:**
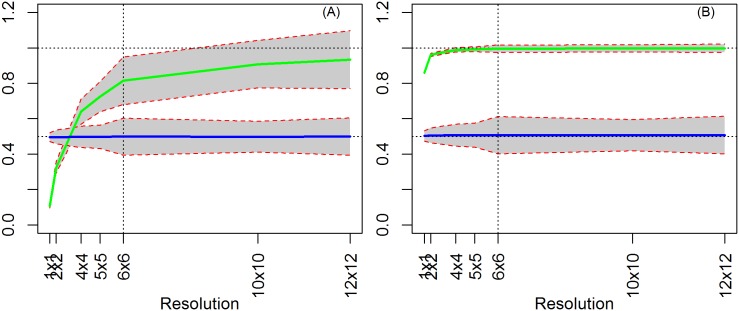
OLS Results (T contiguous, ρ = 0). (A) *X* = *W*^2^*K* and true level = 6x6 resolution. (B) *X* = (1 –πW)*K* and true level = 6x6 resolution.

When we introduce spatial correlation to the independent variable X, we find that even running the regression at the true level, the estimates of *β* are biased, distinct from Model 1 which had an aspatial random X (compare Fig 7A versus Fig 6B). When X is random, the added noise creates imprecision in the variable’s value but its overall distributional structure is maintained, since it is random. Conversely, when X is spatially correlated, besides the previous effect the added noise impacts X’ structure (spatial process) exacerbating its imprecision. As we aggregate, however, the spatial component of X becomes less important for the estimation (since there are fewer observations), leading to a convergence towards its true value.

Note that the bias in the estimates of *β* are driven by the added ‘noise’ in the data due to disaggregation. This effect is more clearly seen when we increase the amount of heterogeneity in the disaggregation procedure, thus introducing more noise, leading to an increased bias in the estimate of *β* (S1 Fig).

As in Model 1, aggregation may smooth away some of the noise if it follows the underlying spatial process of the variable. In our case, the noise is not completely mitigated because aggregation did not follow the spatial process in X (Fig 7A). In comparison, Fig 7B shows the case in which our aggregation procedure more closely follows the spatial pattern of X. However, even with an inadequate aggregation procedure, at higher levels of aggregation, the errors in variables phenomenon is reduced. Finally, as before, aggregation increases the standard errors.

### 4.3. Model 3: OLS with Discrete Measures of (Random) Treatment

The previous models illustrate that while too much aggregation can lead to inefficient estimates, it might help reduce bias in the case of a spatially correlated X. Next, we show that with discrete measures of treatment, common in the conservation literature [1], aggregation can also create bias. To observe this effect, consider the simple scenario when treatment is randomly distributed, and both X and T are random. While this situation does not arise frequently in a conservation setting, one might imagine the situation where one wants to observe the effect of varying levels of enforcement, and that enforcement is distributed randomly over space. Similarly, one might imagine a random set of privately-owned parcels being contributed to a land trust.

**Fig 8:**
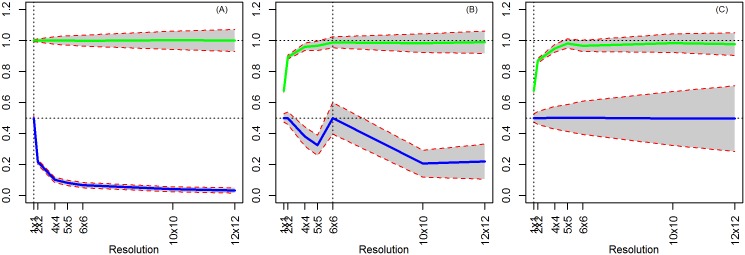
OLS Results (X random, T random, ρ = 0). (A) True level = 1x1 resolution (pixel level) and discrete aggregation of treatment. (B) True level = 6x6 resolution and discrete aggregation of treatment. (C) True level = 6x6 resolution and continuous aggregation of treatment.

Coefficient estimates on X and T are unbiased when estimated at the true level (Fig 8A and 8B). At higher levels of aggregation, the estimate of the treatment effect is attenuated because of the increased noise in its measure. This result again flows from the *errors in variables* problem. Because T is a discrete variable, at higher levels of aggregation, treated pixels are combined with untreated pixels and are allocated either to treatment or not, adding error to the true measure of T. If instead one uses the percent of pixels that are treated as a continuous measure of treatment, this attenuation bias disappears (Fig 8C).

At lower levels of aggregation, the estimate of the treatment effect is unbiased when the true level is perfectly divisible by the unit of analysis. Thus, if the true level is at the 4x4 resolution and the analysis is at the 2x2 resolution, then the treatment estimate is unbiased. If the true level is not perfectly divisible by the unit of analysis, the allocation of treatment to parcels is imprecise. We essentially face the same problem as we do with aggregation, that the noise in the measure of T biases the estimate of the treatment toward zero. This phenomenon is related to the zoning effect, since our unit measure does not perfectly overlap the true data. Note that imperfect allocation to treatment, even at the pixel level, is highly likely in practice since the scenario of perfect treatment allocation would be rare in the real world. Thus, one would expect some attenuation bias when using discrete measures of treatment. Also, at lower levels of aggregation, as in Model 1, we observe that the estimate of the coefficient on X is attenuated towards zero.

### 4.4. Model 4: OLS with Correlated Covariates

We next test the accuracy of our estimates when T is a function of X. We emulate this relationship by allocating treatment to those parcels with high values of X, where X is spatially correlated. This situation most closely resembles how many protected areas are created since criteria for inclusion in a protected area usually include spatially correlated characteristics such as ecosystem type or elevation.

**Fig 9:**
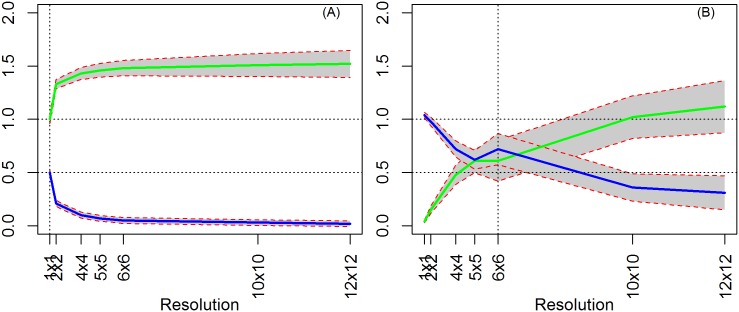
OLS Results (X correlated, T dependent on X, ρ = 0). (A) True level = 1x1 resolution (pixel level). (B) True level = 6x6 resolution.

Now, we retain the same problem with X as in the previous models in that imprecise measures of X at levels of aggregation lower than the true level result in bias. However, biases of the coefficients on treatment and X interact. Two countervailing forces act to bias the coefficient on X. The first is the same as in Models 1 and 2: errors at lower levels of aggregation drive the coefficient estimate toward zero. The other bias concerns the misestimation of the coefficient on T, which is correlated with X. The underestimation of the coefficient on T means that some of the variation in outcome truly associated with T is incorrectly attributed to X, resulting in overestimation of the coefficient on X. This result differs from what we observed in the second scenario where this interaction does not occur. The degree of overestimation is worse when the bias on *γ* is also worse, as can be seen at higher levels of aggregation when the true level is at the pixel level (Fig 9A and 9B). Thus, the larger the correlation between X and T, a discrete variable which is increasingly distorted with aggregation, the more that aggregation biases is the estimates of the coefficient on X.

Unlike earlier models in which the coefficient on T was always underestimated, the correlation between X and T implies that the treatment estimates may be either under or overestimated. The bias on the estimated treatment effect goes from positive (or zero) to negative as we increase the level of aggregation. The degree of negative bias is worse at higher levels of aggregation for the same reason as discussed in Model 3: as treated parcels are aggregated with some untreated parcels, the allocation to treatment is committed with error, inducing an error in variables problem, resulting in an increasingly attenuated coefficient estimate. At low levels of aggregation, the measure of T is relatively precise while the measure of X is imprecise, implying that more explanatory power is levied on T, resulting in an overestimate of the treatment effect. At higher levels of aggregation, X is now measured more precisely than T and this situation reverses, resulting in an underestimation of the treatment effect.

Our model produces unbiased estimates at the true level when the true level and the analysis occur at the pixel level (Fig 9A) but produces biased estimates above the pixel level (Fig 9B) even if our analysis uses the same scale as the true data generating process. As above, this is a result of the noise added to the “observed data” at the pixel level, which then gets transferred with aggregation. This bias is evident in that at the vertical dashed line in Fig 9B, the estimated coefficient on T coefficient is around 0.75 (as compared with a true coefficient of 0.5) and the estimated coefficient on X is around 0.6 (in contrast to a true value of 1).

### 4.5. Model 5: OLS with Spatial Lag

Finally, we revisit the above models with an added spatial lag in the dependent variable Y. At *ρ* = 0.5, the true marginal effect of T should be 1 and of X should be 2. With clustered treatment (Fig 4A), the primary difference from the scenario without a spatial lag in Y is that the estimated treatment effect is slightly biased downward from the true marginal effect of 1. With other scenarios, we essentially observe the same problems as without the spatial lag in Y, except that even at the true level, the estimated coefficients are not equal to the marginal effects, and move further from those marginal effects as spatial correlation increases. This result is expected since we essentially introduce an *omitted variable bias* with the spatial lag. Moreover, the addition of a spatial lag to spatially autocorrelated covariates induces even more bias relative to the case with no spatial lag in Y. Thus, with the spatial lag in Y, one obtains biased estimates even at the appropriate level of aggregation. These effects are demonstrated in S3 Fig.

### 4.6. Models 1–5 using Different Estimation Strategies

#### Spatial Two-Stages Least Squares (STSLS)

**Fig 10:**
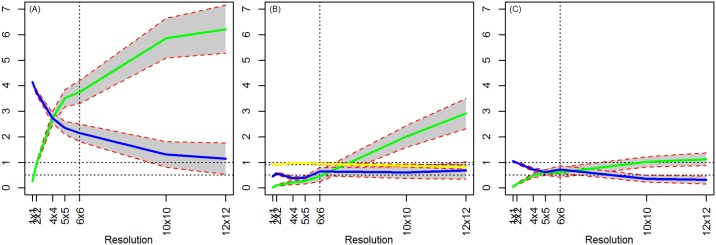
OLS versus STSLS results (true level = 6x6 resolution). (A) OLS result (X correlated, T = f(X), ρ = 0.9). (B) STSLS result (X correlated, T = f(X), ρ = 0.9). (C) OLS result (X correlated, T = f(X), ρ = 0).

A few authors have used a spatially-lagged regression to estimate the effect of conservation programs when they suspect that the outcome follows a spatial-lag process [17,24]. In one form of this regression, the spatial lagged values of Y are instrumented using spatially lagged values of the independent variables. Using the Spatial Two-Stages Least Squares (STSLS) approach, we attempt to solve the omitted variable issue. By comparing Fig 10A (OLS) and Fig 10B (STSLS), the omitted variable bias is well mitigated at and below the true level. Nonetheless, at higher levels of aggregation, although attenuated, the bias is still substantial (Fig 10B and 10C). This bias results from the aggregation process that distorts the structure of the spatial process in Y, so that the spatial weights matrix does not accurately reflect the true spatial process of Y, reducing the explanatory power of the lagged Y covariate. Notice that this phenomenon is similar to what occurs to the estimates on discrete treatment and spatially correlated X during aggregation.

#### Difference-in-Difference

Another common technique in the conservation literature is the use of difference-in-differences approach with panel data to estimate the impact of conservation programs [25]. To study the impact of scale on difference-in-difference estimations, we generate a panel dataset of the form:

$$Y_{it}=\beta_{0}+\beta_{1}X_{it}+\rho WY_{it}+\gamma T+\;v_{it}$$

$$v_{it}=\varepsilon_{i}+\varepsilon_{t},\;\varepsilon_{i}\;\sim N\left(0,\;1\right),\;\varepsilon_{t=1}\sim N\left(0,\;1\right),\;\varepsilon_{t=2}\sim N\left(1,\;0.5\right)$$

**Fig 11:**
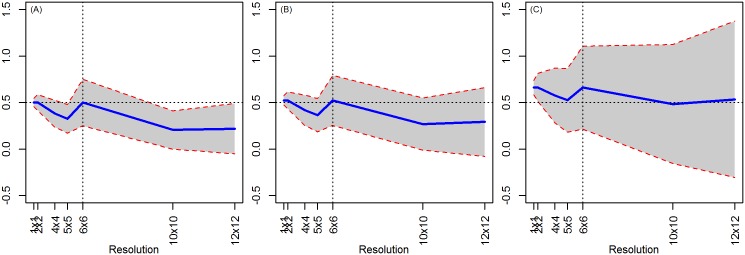
Difference-in-Difference results (X and T random and true level = 6x6 resolution). (A) ρ = 0. (B) ρ = 0.5. (C) ρ = 0.9.

As with the OLS analysis, estimates are unbiased at the true level but changes in scale induce bias while standard errors increase with aggregation (Fig 11C). Greater spatial heterogeneity at lower aggregation levels merely increases the standard errors.

By imposing a spatial process in the dependent variable, we observe an increasing bias in the estimated treatment coefficient at the true level. However, as the spatial dependence increases, it tends to smooth out this pattern (Fig 11B; also, Fig 11A shows results with no spatial lag, and the spatial lag increases as one moves right). Standard errors tend to increase with increasing spatial dependence. If treatment is clustered, constant bias is observed. The existence of spatial autocorrelation in X increases the amount of bias induced (S7 Fig).

#### Do alternative estimation methods partially solve the MAUP?

**Fig 12:**
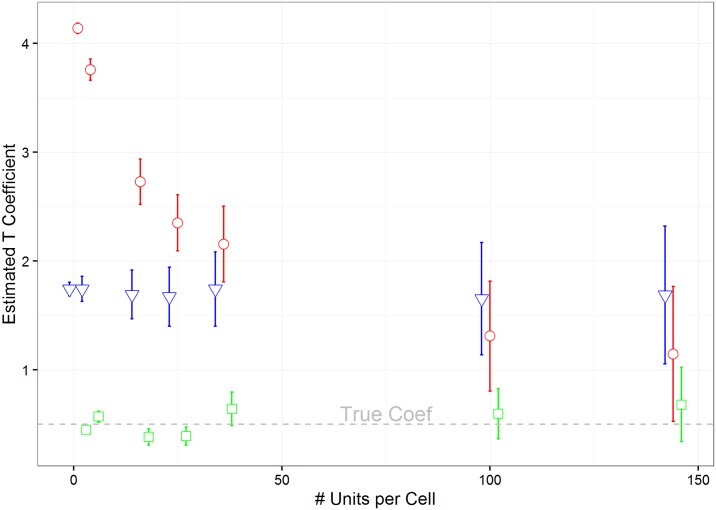
Comparison of different estimation methods (X correlated, T dependent on X, ρ = 0.9). OLS (circles), STSLS (squares), DID (triangles). True level = 6x6 resolution, with 1,000 simulations.

Although techniques other than OLS can mitigate some dimension of bias in the MAUP scale effect, they cannot fully eliminate the issues. However, between OLS, DID and STLS, STLS mitigates the most bias as it solves the omitted variable issue and counterbalances the error in variables bias (Fig 12).

## 5. Discussion

In this paper we examine how choices of scale impact estimates of treatment effects when evaluating conservation programs designed to protect land cover. We take away several lessons. First, as expected, aggregation generates efficiency loss. When all variables are continuous and randomly generated, and there are no spatial processes nor correlation of covariates, then aggregation generates efficiency loss, but not bias. This result is highlighted in previous literature on the MAUP [e.g., 5, 14]. Aggregation can also generate bias when it combines treated and untreated areas, making the measure of treatment less precise and biasing estimates towards zero.

Second, perhaps more surprisingly, we find that selecting a scale that is too small can be problematic. When we examine a phenomenon at a finer resolution than the relevant scale, the noise introduced in the covariates generates an *error in variables* bias, which is carried throughout the different levels of aggregation. Aggregation tends to mitigate the bias driven by errors in variables as it smoothes out the noise. However, this reduction in error only holds for continuous variables. Discrete variables and spatial lags introduce another source of error in variables bias as aggregation distorts their spatial structure. Hence, we show that the MAUP creates both bias and inefficiency in the estimates. This finding differs from the previous literature: past research examining efficiency problems has focused exclusively on aggregation, not imprecision generated from disaggregation.

In sum, we show that an areal unit that is too big can be a problem and an areal unit that is too small can also be a problem. What scale might Goldilocks’ call “just right”? Ideally, one would like to choose a unit of observation that corresponds with the scale that determines the variable of interest. As noted earlier, this choice is difficult because the data generating process that drives the dependent variable may not be readily apparent or may occur at multiple scales simultaneously.

Perhaps most concerning, even if one can identify the scale at which the data generating process occurs, and one has a well-specified statistical model, scale may still be a problem. If two variables are highly correlated but the error in variables affect their precision differently, we observe increasing bias in the more precise variable as OLS compensates the loss in explanation power of the other. In this case, choosing the unit of analysis at the appropriate scale will not be sufficient to eliminate bias. Furthermore, the bias may overestimate or underestimate the treatment effect. Thus, even a scalar choice that is ‘just right’ can produce wrong estimates. Therefore researchers should carefully consider how treatment may correlate with X variables, and test sensitivity to this problem.

In short, even with relatively simple simulated spatial data, we find that the MAUP can generate a complex set of biases. Spatial processes further complicate the issue. By ignoring an existing spatial lag process, we introduce another layer of bias on top of the previous ones driven by omitted variables.

Many alternative estimation methods have been used to precisely estimate treatment of conservation programs. We show that biases persist throughout multiple methods, including using a difference-in-difference approach with fixed effects although, the spatial two stages least squares seems a promising alternative to mitigate some of the estimation issues for treatment.

In most spatial datasets used in conservation, land use or other data from satellite imagery, researches should expect to encounter the spatial correlations and other issues described above. These results are a cautionary tale for anyone using satellite imagery or other spatial data, especially since these challenges cannot be addressed with existing methods. Nevertheless, we offer a few possible best practices and questions to consider when deciding the appropriate scale for undertaking this analysis.

First, the key challenge for researchers is to choose the relevant scale for the process being modeled. Regardless of the precision available for explanatory variables, researchers should prioritize the relevant scale for the dependent variable. High resolution data may be useful in observing phenomenon in great detail, but researchers should not assume more resolution is always better, because the highest level of resolution is not necessarily the appropriate unit of analysis. Understanding how much to aggregate high resolution data is crucial to generate unbiased results. Dealing with the problem of working at too fine a resolution is relatively new for the field. Our results show that selecting an areal unit that is too small can be equally or more problematic than scales that are too big. Until now, most of the concern has been about overcoming the ecological fallacy and dealing with data that are too coarse. We find that with new higher resolution data, researchers must devote more time to thinking through the most appropriate scale and unit of analysis for their study site, conservation practice and the variable being modeled. When feasible, one would ideally conduct the analysis at multiple scales to test for bias [7].

Intermediate scales may most accurately explain land use changes in deforestation models, while reforestation models may occur at a smaller scale. These intermediate scales could potentially be at a sub-parcel level, but not fully exploit the 1 m or sub meter spatial data increasingly available. When reviewing the scale used by research in the existing literature on deforestation, we sense that often there is room for finer scale resolutions to be used to improve the analysis, however we should not assume that smallest resolution available will provide the most accurate results. Obtaining data at a smaller pixel size is unambiguously beneficial in that one can always aggregate up to larger units. At the same time, having obtained data at a finer resolution does not mean one should conduct the analysis using a smaller analytical unit. The use of a smaller unit of analysis is important to avoid combining different land uses, and property owners and treatment areas in the same areal unit, however pixel level analysis is likely to generate noise that will distort the results.

Second, researchers should examine the degree and nature of the spatial correlation among their variables to determine how errors in one variable might affect the estimates of the effect of the others. We observed that when independent variables (X) are spatially correlated to treatment, all of the explanatory power is attributed to one or the other, causing extreme over and underestimates.

Third, we would advise future research to use continuous rather than discrete measures of treatment for each areal unit (i.e., percent protected instead of protected versus not protected) Similarly, discrete measures of outcomes (forest versus not forest) are likely to induce inaccuracy, which increases at higher levels of aggregation. Using a continuous measure of treatment mitigates against the increased problem of errors in variables associated with aggregation. Adopting continuous measures of treatment and outcomes is an easy step researchers can take to protect themselves from the problems discussed above.

Last, this research also suggests that the conservation literature may benefit from the integration of lessons developed by hierarchical models that have integrated processes operating at multiple scales [26].

Perhaps most importantly, our results suggest a possible direction for future research that could deliver some simple solutions. Future work includes developing a method for determining bounds on estimates that take aggregation and spatial correlation into account, and the development of diagnostic tests to determine the degree to which these issues may be problematic. Such methodological progress will be essential to mainstream impact evaluation in nature conservation [27].

## Supporting Information

S1 FigSensitivity analysis (X correlated, T contiguous, ρ = 0).True level = 6x6 resolution. (A) Disaggregation using $\sigma_{d}^{2}=0.01$. (B) Disaggregation using $\sigma_{d}^{2}=1$. (C) Disaggregation using $\sigma_{d}^{2}=5$.(TIF)

S2 Fig OLS Results (X correlated, ρ = 0). (A) T = Contiguous and true level = 6x6 resolution. (B) T = Random and true level = 6x6 resolution.
(TIF)

S3 Fig OLS results with different spatial lags and true level = 6x6 resolution. (A) X correlated, T contiguous, ρ = 0.5. (B) X correlated, T contiguous, ρ = 0.9. (C) X random, T random, ρ = 0.5. (D) X random, T random, ρ = 0.9. (E) X correlated, T = f(X), ρ = 0.5. (F) X correlated, T = f(X), ρ = 0.9.
(TIF)

S4 Fig Effects of additional complexity in the data on OLS results (true level = 6x6 resolution). (A) Model 1: No Noise (Y random, X random, T contiguous). (B) Model 1b: Noisy Dataset (Y random, X random, T contiguous). (C) Model 2: Spatial Process on X (Y random, X s.a., T contiguous). (D) Model 3: Discrete Aggregation (Y random, X random, T random). (E) Model 3b: Binary Aggregation (Y random, X s.a., T random). (F) Model 4: Covariate Correlation (Y random, X s.a., T = f(X)). (G) Model 5: Spatial Lag (Y s.a., X s.a., T = f(X)).
(TIF)

S5 Fig Summary of Estimated Coefficient on X from OLS Results. True level = 6x6 resolution.
(TIF)

S6 Fig Summary of Estimated Coefficient on T from OLS Results. True level = 6x6 resolution.
(TIF)

S7 Fig Difference-in-Difference results when X correlated, T = f(X) and true level = 6x6 resolution. (A) ρ = 0.5. (B) ρ = 0.9.
(TIF)
