# Supplementary figures and images for: Goldilocks and the Raster Grid: Selecting Scale when Evaluating Conservation Programs
Source: PLoS One. 2016 Dec 22;11(12):e0167945. doi: 10.1371/journal.pone.0167945 (PMC5179101; doi:10.1371/journal.pone.0167945)

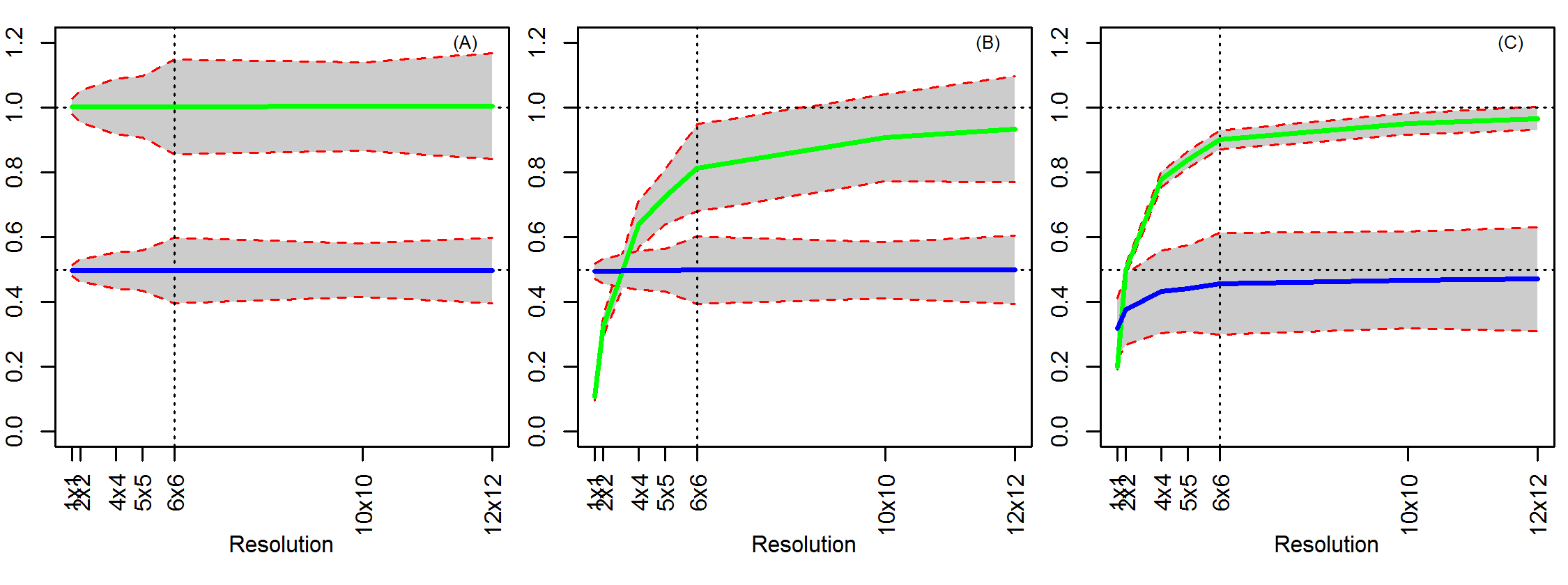

Supplement: S1 Fig — True level = 6x6 resolution. (A) Disaggregation using σd2=0.01. (B) Disaggregation using σd2=1. (C) Disaggregation using σd2=5. (TIF) [file pone.0167945.s001.tif]

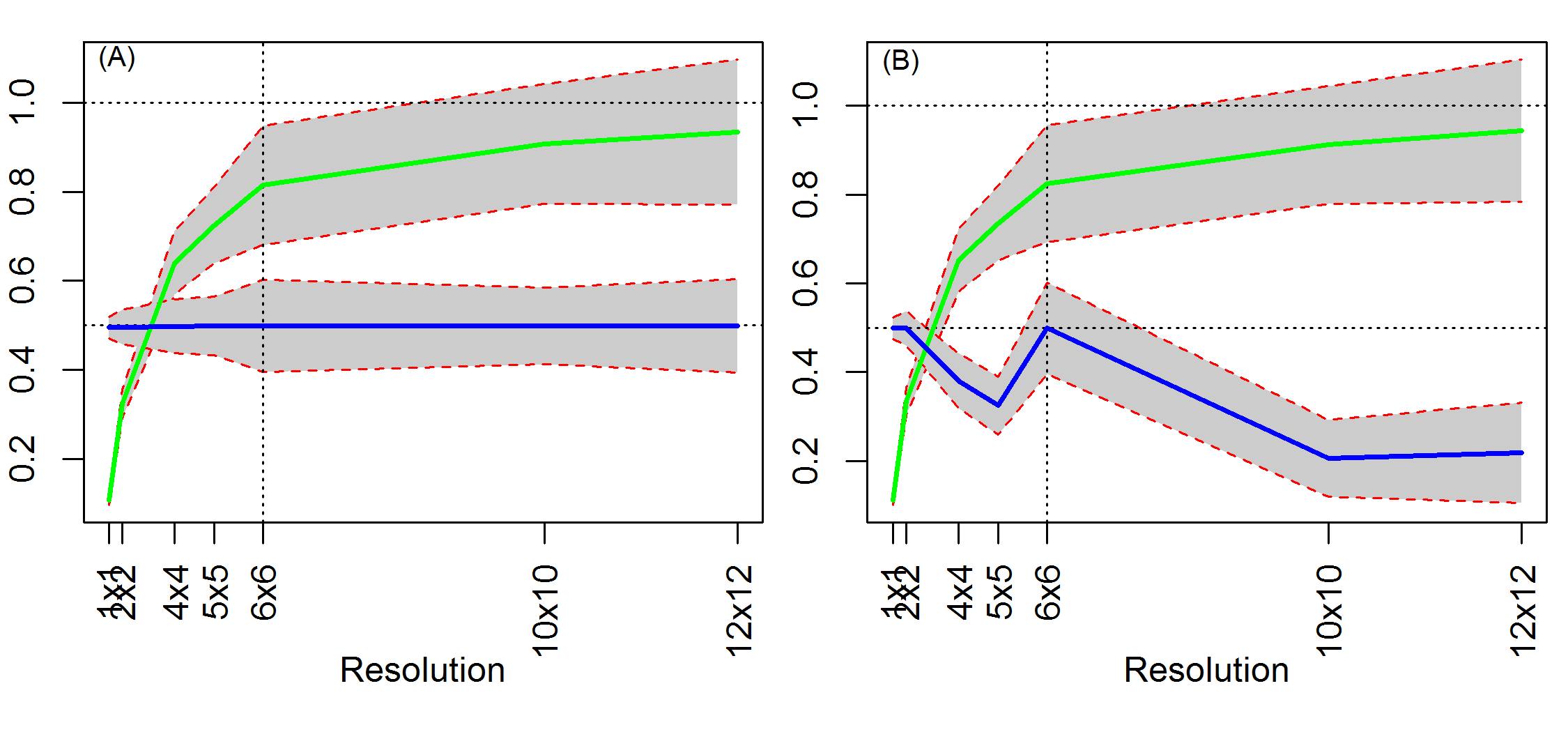

Supplement: S2 Fig — (A) T = Contiguous and true level = 6x6 resolution. (B) T = Random and true level = 6x6 resolution. (TIF) [file pone.0167945.s002.tif]

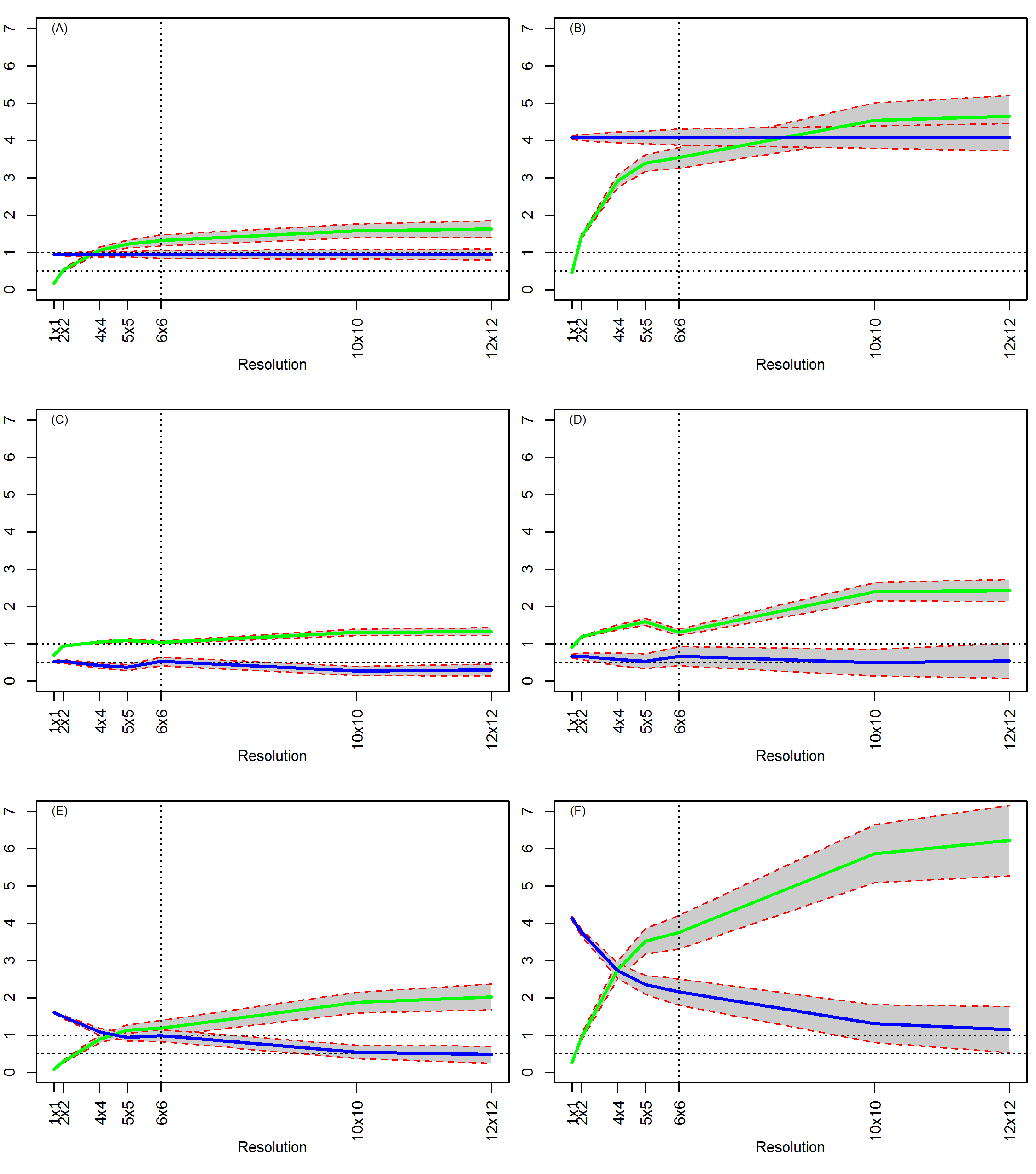

Supplement: S3 Fig — (A) X correlated, T contiguous, ρ = 0.5. (B) X correlated, T contiguous, ρ = 0.9. (C) X random, T random, ρ = 0.5. (D) X random, T random, ρ = 0.9. (E) X correlated, T = f(X), ρ = 0.5. (F) X correlated, T = f(X), ρ = 0.9. (TIF) [file pone.0167945.s003.tif]

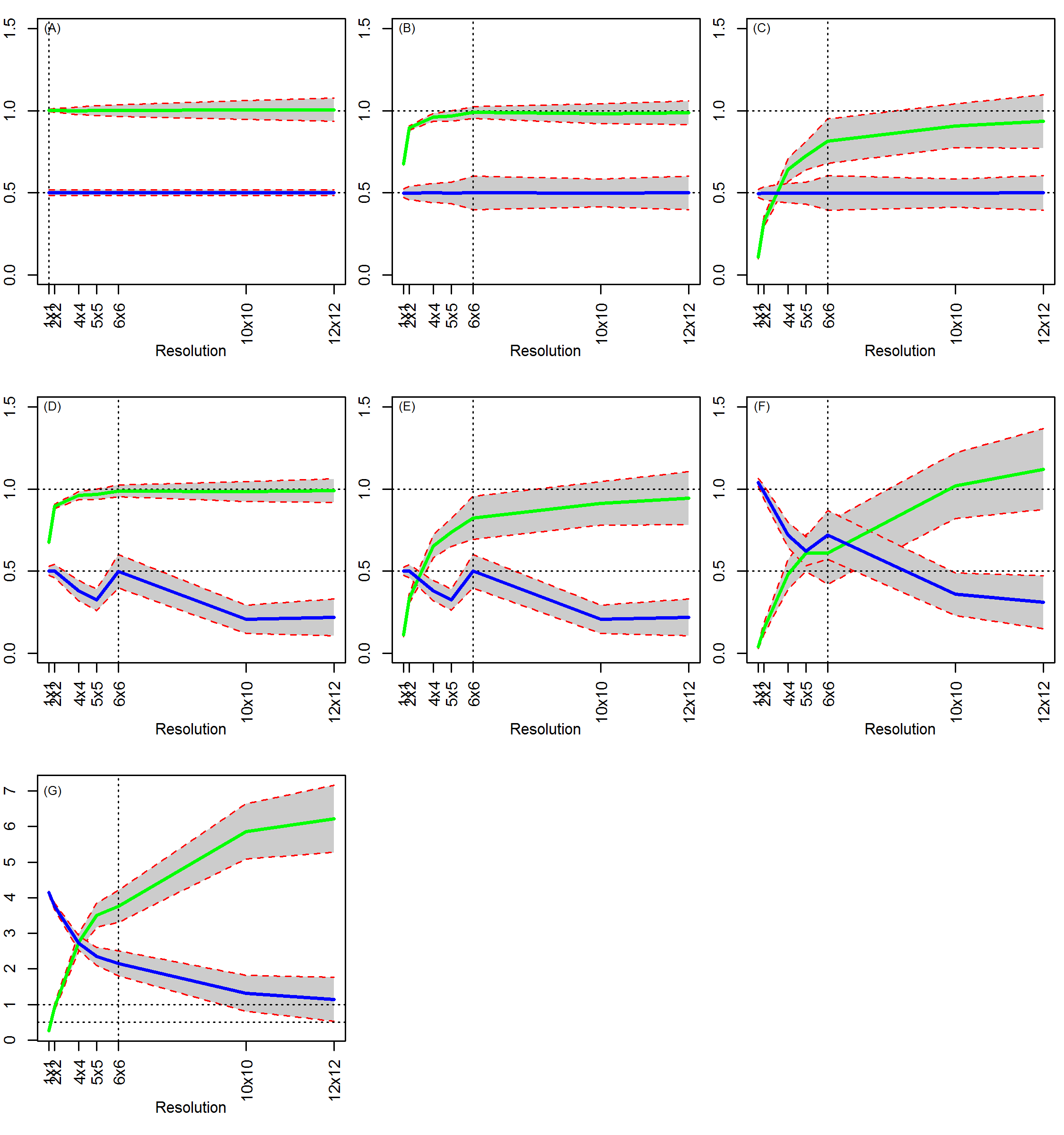

Supplement: S4 Fig — (A) Model 1: No Noise (Y random, X random, T contiguous). (B) Model 1b: Noisy Dataset (Y random, X random, T contiguous). (C) Model 2: Spatial Process on X (Y random, X s.a., T contiguous). (D) Model 3: Discrete Aggregation (Y random, X random, T random). (E) Model 3b: Binary Aggregation (Y random, X s.a., T random). (F) Model 4: Covariate Correlation (Y random, X s.a., T = f(X)). (G) Model 5: Spatial Lag (Y s.a., X s.a., T = f(X)). (TIF) [file pone.0167945.s004.tif]

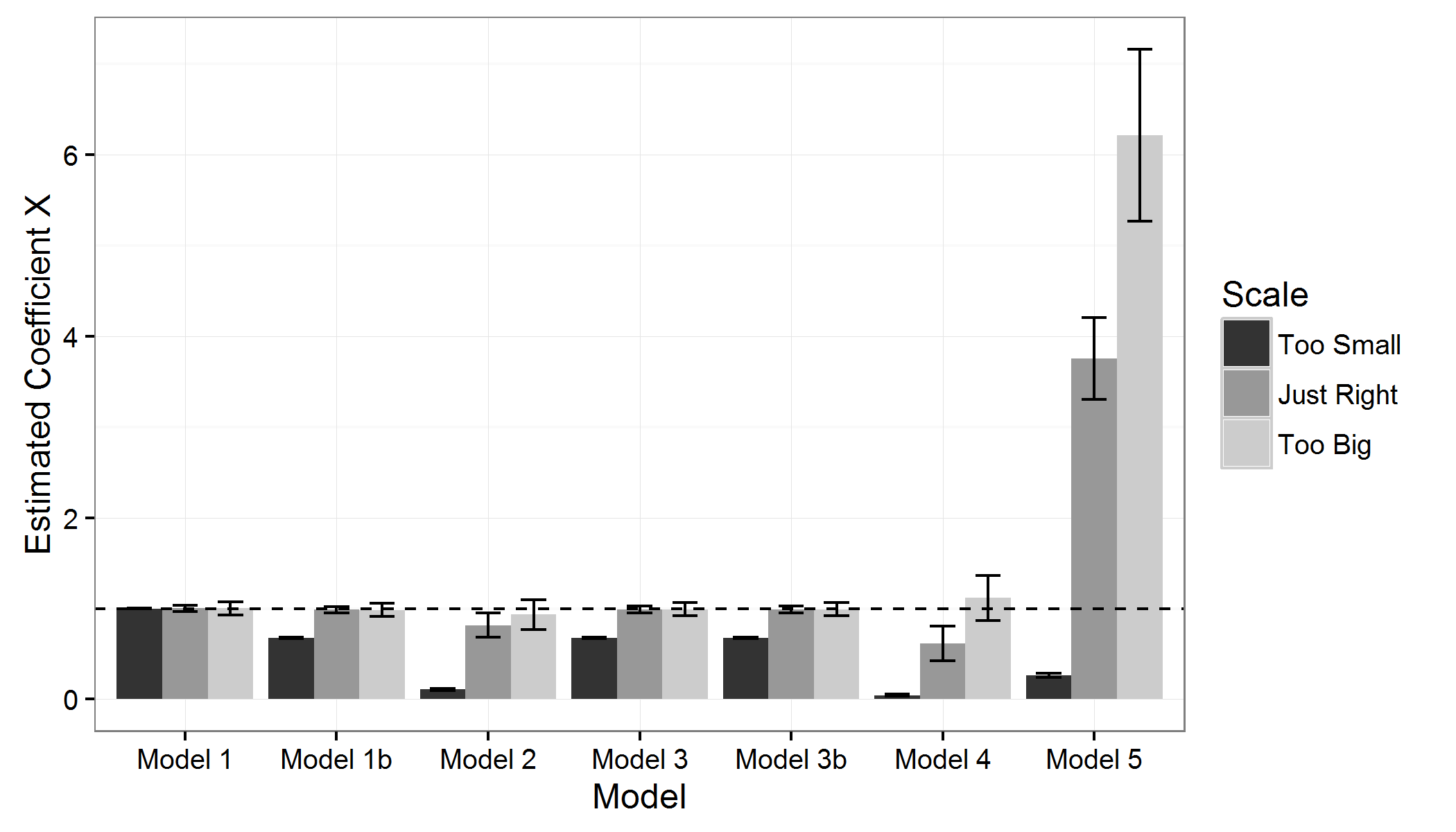

Supplement: S5 Fig — True level = 6x6 resolution. (TIF) [file pone.0167945.s005.tif]

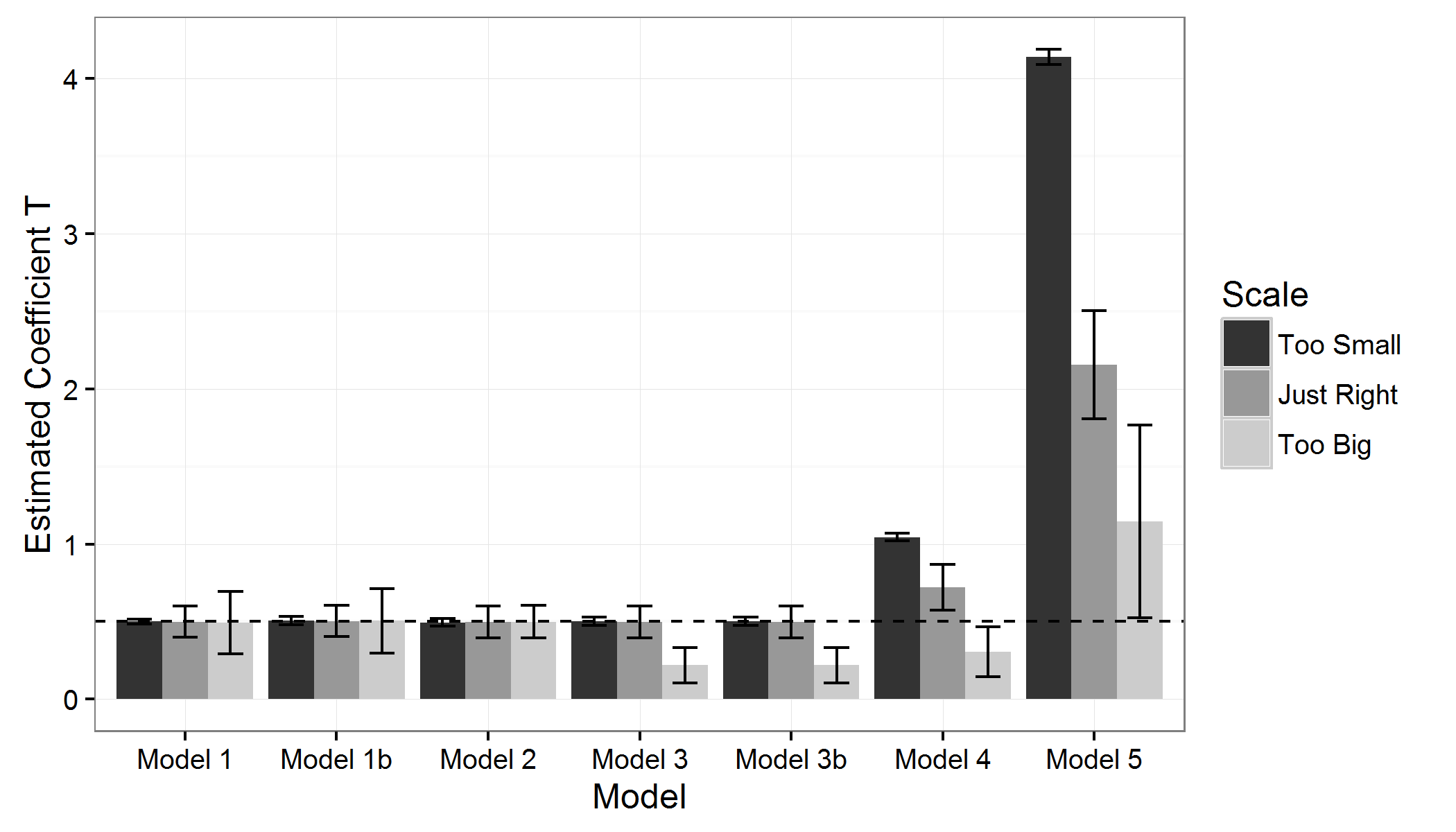

Supplement: S6 Fig — True level = 6x6 resolution. (TIF) [file pone.0167945.s006.tif]

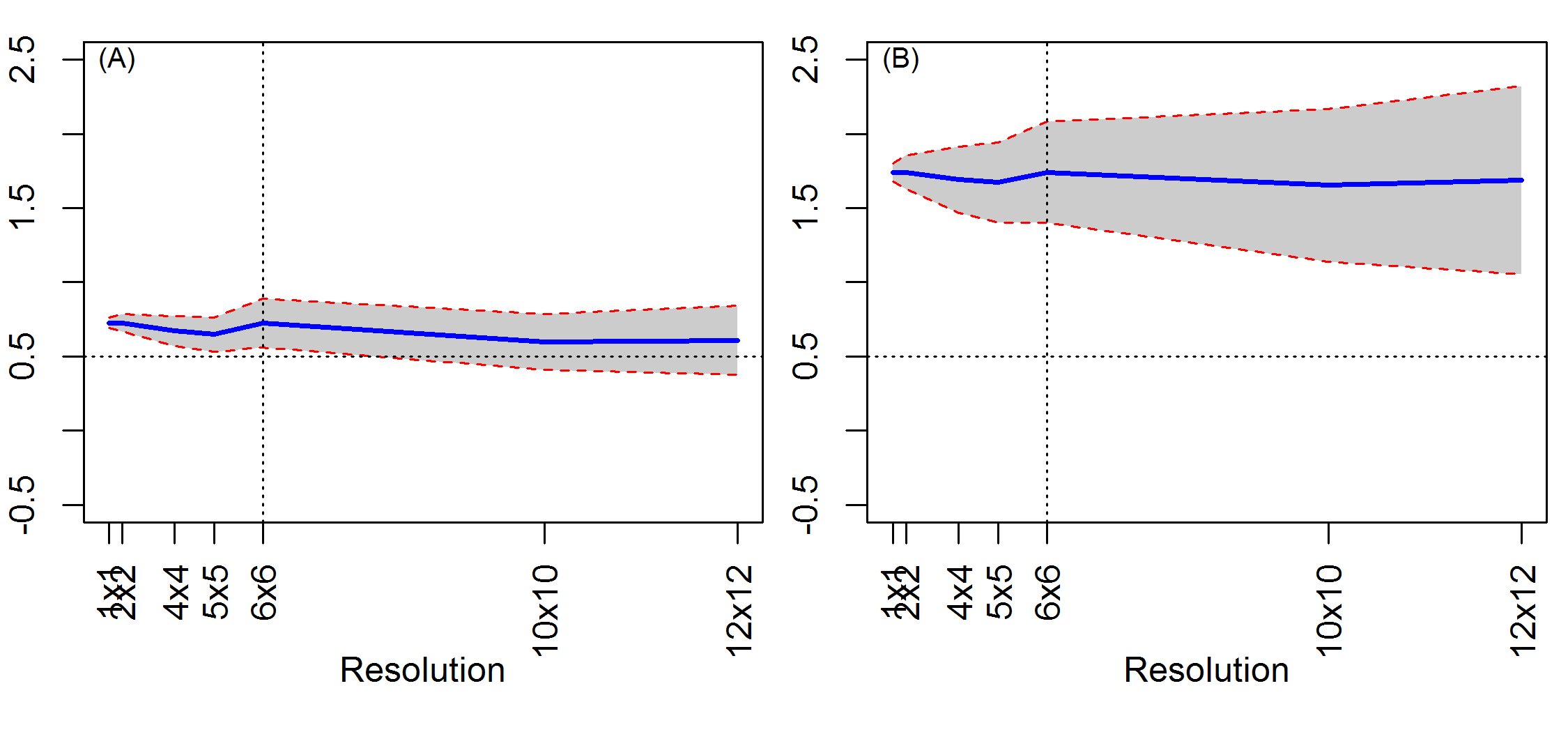

Supplement: S7 Fig — (A) ρ = 0.5. (B) ρ = 0.9. (TIF) [file pone.0167945.s007.tif]
